# Improvement of crAssphage detection/quantification method and its extensive application for food safety

**DOI:** 10.3389/fmicb.2023.1185788

**Published:** 2023-05-15

**Authors:** So-Young Lee, Jihye Yang, Ju-Hoon Lee

**Affiliations:** ^1^Department of Food Science and Biotechnology, Institute of Life Sciences and Resources, Kyung Hee University, Yongin, Republic of Korea; ^2^Department of Agricultural Biotechnology, Seoul National University, Seoul, Republic of Korea; ^3^Center for Food and Bioconvergence, Seoul National University, Seoul, Republic of Korea; ^4^Research Institute of Agriculture and Life Sciences, Seoul National University, Seoul, Republic of Korea; ^5^Department of Food and Animal Biotechnology, Seoul National University, Seoul, Republic of Korea

**Keywords:** crAssphage, quantitative real-time PCR, human fecal contamination, rapid detection, food safety

## Abstract

Water-borne diseases are usually caused by the fecal–oral transmission of human fecal pathogens. Traditionally, coliforms and enterococci are widely used as indicator bacteria, but they do not allow to differentiate between human and animal fecal contamination. Owing to its presence only in the human gut environment, crAssphage has been suggested as an alternative indicator of human fecal contamination to overcome the above challenges. In this study, 139 human and 89 animal fecal samples (e.g., chicken, cow, dog, pig, pigeon, and mouse) were collected. For the rapid detection of human crAssphage in fecal samples, quantitative real-time PCR (qPCR) was performed using five different oligonucleotide primer/probe combinations. These included three previously reported oligonucleotide primer/probe combinations (RQ, CPQ056, and CrAssBP) and two newly developed combinations (ORF00018-targeting CrAssPFL1 and ORF00044-targeting CrAssPFL2). The detection rate (crAssphage-positive rate) in human fecal samples were 23.0, 30.2, 28.8, 20.1, and 30.9%, respectively, suggesting CrAssPFL2 showed the highest detection rate. Furthermore, the lowest copy numbers (436.16 copy numbers) could be detected using the CrAssPFL2 combination. Interestingly, no difference in crAssphage detection rates was found between healthy people and intestinal inflammatory patients. As expected, no crAssphage was detected in any animal fecal samples, indicating its human specificity. Furthermore, qPCR analysis of sewage samples collected from five different sewage treatment plants revealed that they were all contaminated with 10^5.71^ copy numbers/mL of crAssphage on average. The simulation test of crAssphage-contaminated food samples also confirmed that the detection limit was from 10^7.55^ copy numbers of crAssphage in foods. Therefore, the newly developed and optimized qPCR would be useful for the sensitive detection of crAssphage while identifying the source of human fecal contamination.

## Introduction

Human fecal contamination of drinking, recreational, and environmental waters is generally due to sewage outfalls, leaking septic tanks, and urban and agricultural runoff (Geary and Davies, 2003; Fewtrell and Kay, 2015). Enteric diseases as associated with exposure to contaminated water include *Vibrio* spp.*, Salmonella* spp. and *Escherichia coli*, and norovirus (Bryan, 1977; Nel and Markotter, 2009; Fazal-ur-Rehman, 2019). According to the World Health Organization, enteric diseases account for approximately 4.1% of the total daily global burden of disease while causing approximately 1.8 million human deaths annually (Pathak, 2015). In addition, the US Centers for Disease Control and Prevention reported approximately 7.15 million cases of water-borne diseases annually, resulting in 601,000 emergency department visits, 118,000 hospitalization, and 6,630 deaths with economic loss of $3.33 billion US (DeFlorio-Barker et al., 2021). Therefore, prevention of contamination of water with human waste is important and methods that detect all classes of human enteric pathogens would be ideal.

To date, fecal indicator bacteria (FIB) such as enterococci and *Escherichia coli*, have been primarily used to determine fecal contamination. However, because these organisms are present in human as well as animal fecal material and even in the environment, FIBs are not efficient indicators for human fecal pollution (Payment and Locas, 2011). To overcome this limitation, new microbial source tracking (MST) markers have been developed including human intestinal bacteria (*Bacterioidales, Bifidobacteirum, Enterococcus, Lachnospiraceae, E. coli*, etc.), human intestinal archea (*Methanobrevibacter smithii*), viruses (F^+^ RNA coliphage, pepper mild mottle virus (PMMoV), HPyVs), and human mitochondrial DNA (Harwood et al., 2014). Although most MST marker genes (pathogen-specific genes or 16S rRNA genes) are associated with humans fecal pollution they are not 100% specific.

In 2014, crAssphage was discovered in human fecal samples by metagenomics (Dutilh et al., 2014), but it was not successfully isolated from human fecal samples. In 2018, Shkoporov et al. successfully isolated and characterized crAssphage ΦcrAss001, the first human-specific crAssphage, and showed that it infects the human gut bacterium *Bacteriodes intestinalis* (Shkoporov et al., 2018). Subsequently, a second crAssphage, ΦcrAss002, was isolated from *B. xylanisolvens* (Guerin et al., 2021). Metagenomic analysis in sewage samples showed a significantly higher abundance of crAssphage (>6,000 reads mapped) compared to other viral MST markers such as PPMoV and HPyV (<1,000 reads mapped) (Stachler and Bibby, 2014). To detect crAssphage in human fecal samples, several research groups developed specific oligonucleotide primer/probe combinations, CPQ056, CPQ064 (Stachler et al., 2017; Ahmed et al., 2018, 2019; Zhang et al., 2020), RQ (Cinek et al., 2018), and the combination of TN201 (forward primer), TN203 (reverse primer), and a probe TN202 (Park et al., 2020). These developed crAssphage-targeting qPCR oligonucleotide primer/probe combinations were also used for the detection and determination of human fecal contamination in various environmental samples (Ahmed et al., 2018, 2019; Kongprajug et al., 2019; Li et al., 2021; Nam et al., 2022). However, it is still required to develop and evaluate an improved new combination for optimum detection and determination of crAssphage in the human gut. In addition, although each combination was evaluated in the human fecal samples, it is still necessary to compare the detection efficiency and accuracy of these combinations within the same fecal samples. Furthermore, no studies have analyzed the correlation between the abundance of crAssphage and human intestinal disease.

Two new oligonucleotide primer/probe combinations, CrAssPFL1 and CrAssPFL2, for the detection of crAssphage were developed based on the complete genome sequences of uncultured crAssphage. Their human specificity and detection efficiency were verified with various human and animal fecal samples from South Korea. In addition, these new combinations were compared and evaluated with previously developed crAssphage-targeting combinations, CrAssBP (Park et al., 2020), CPQ056 (Stachler et al., 2017), and RQ (Cinek et al., 2018) using the same fecal samples. Furthermore, the CrAssPFL2 primer/probe combination was selected to study the correlation between the abundance of crAssphage and irritable bowel syndrome (IBS)/colorectal cancer (CRC) between healthy human and patient fecal samples in South Korea. Therefore, this study would be useful for the rapid detection of crAssphage and accurate determination of human fecal contamination with these newly developed and optimized primer/probe combinations. It would also extend our knowledge of the relationship between crAssphage and human intestinal diseases.

## Materials and methods

### Primers and probes

Table 1 lists the sequences of crAssphage-targeting primers and primer/probe combinations. For conventional PCR, the DNA sequences of a For/Rev18-F primer set were obtained from the previous study (Liang et al., 2016), and CrAssORF24 and CrAssORF44 primer sets were designed using Online PCR Primers Designs Tool (GenScript, United States). For qPCR, the DNA sequences of CrAssBP, CPQ056, and RQ primers and probe combinations were acquired from each previous study (Stachler et al., 2017; Cinek et al., 2018; Park et al., 2020). The CrAssPFL1 and CrAssPFL2 oligonucleotide primers and probe combinations were designed using the real-time PCR (TaqMan) Primer and Probes Design Tool (GenScript). All primers and probes were synthesized and purified using BIONICS (South Korea).

**Table 1:** Primers and probes designed and used in this study.

Primer/probe name	Target	Primer and probe sequences^a^		Size (bp)	Position	Reference
Primers for qPCR						
CrAssPFL1	ORF00018	Forward	5′-ATG ACC GTC TTG CTG TTC TT-3′	200	488–507	In this study
		Reverse	5′-ATC TGC TTG CAT TCC AGT AA-3′		668–687	
		Probe	FAM-TGC TTC TCA TGT TAT GCG AAG CTC TTG-TAMRA		533–559	
CrAssPFL2	ORF00044	Forward	5′-ACT GGA GAT GAA CCT ACA AGA C-3′	196	26,734–26,755	In this study
		Reverse	5′-TCC AAC TAT CTT TAA TTA CAA CAG C-3′		26,905–26,929	
		Probe	FAM-CCA CAT CCA AGC AAT AGC ATC AGC ACA-TAMRA		26,818–26,933	
CrAssBP	ORF00018	Forward	5′-ATG TWG GTA RAC AAT TTC ATG TAG AAG-3′	193		Park et al. (2020)
		Reverse	5′-TCA TCA AGA CTA TTA ATA ACD GTN ACA ACA-3′			
		Probe	FAM-ACC AGC MGC CAT TCT ACT ACG AGH AC-BHQ1			
CPQ056	ORF00024	Forward	5′-CAG AAG TAC AAA CTC CTA AAA AAC GTA GAG-3′	126		Stachler et al. (2017)
		Reverse	5′-GAT GAC CAA TAA ACA AGC CAT TAG C-3′			
		Probe	FAM-AAT AAC GAT TTA CGT GAT GTA AC-MGB			
RQ	ORF00044	Forward	5′-GGT AAG AAT ATT ACT GAA TAT CCT ACT TG-3′	182		Cinek et al. (2018)
		Reverse	5′-CAA TCA TGT TCA TCA ATA AAY GCT TCA-3′			
		Probe	FAM-ATG ATA TTA ATT ATC TTA CTG GAG ATG AAC CTA CAA GAC AAA C-BHQ1			
Primers for conventional PCR						
For/Rev18-F	ORF00018	Forward	5′-CGGCGGGTTAATCAAAATAGAA-3′	2,420	8,911–8,928	Liang et al. (2016)
		Reverse	5′-GCGGAGAACCCCATTTATTAATAAG-3′		11,310–11,330	
CrAssORF24	ORF00024	Forward	5′-GAA CCT GTT CGT ATC GGT AAG-3′	1,213	14,504–14,524	In this study
		Reverse	5′-GGT ACT AAA ATA GTA CCC AAT CCT C-3′		15,740–15,716	
CrAssORF44	ORF00044	Forward	5′-CAT AGA TAT GAT TCT TTT GCC C-3′	768	26,321–26,342	In this study
		Reverse	5′-CAA GCG TCA CAA CCA TCT-3′		27,071–27,088	

a FAM, Carboxyfluorescein; TAMRA, Carboxytetramethylrhodamine; BHQ1, Black Hole Quencher-1; MGB, Minor groove binder.

### Collection of fecal and sewage samples

A total of 139 human fecal samples were collected: Human participants were reviewed and approved by the Institutional review board (IRB). 37 fecal samples from healthy Korean adult volunteers in their 20s and 30s, 35 fecal samples from IBS children (*n* = 3) and adolescents’ patients (*n* = 32) at the Samsung Seoul Hospital, and 67 fecal samples from CRC patients aged from 18 to 75 at the Seoul St. Mary’s Hospital, Samsung Seoul Hospital, Asan Medical Center, and Seoul National University Bundang Hospital in South Korea. In addition, 89 animal fecal samples were collected: 15 dog fecal samples and 7 pigeon fecal samples (Seoun Sports Park, Incheon, South Korea), 20 pig fecal samples (Jeoneui, Chungnam), 7 cow fecal samples (Asan, Chungnam), 3 chicken fecal samples (Gunwi, Gyeongbuk), and 37 laboratory mouse fecal samples at Dankook University (Cheonan, Chungnam). All fecal samples were transported to the laboratory within 24 h with an ice pack and stored in a deep freezer (Duofreez; Daihan, South Korea) at −80°C. In addition, untreated sewage samples were obtained from the Seongnam Water Quality Restoration Center, Gwangju Sewage Treatment Plant (STP), Bongdam STP, Gyeongan STP, and Opo STP in Gyeonggi, South Korea. A total of 500 mL of each sample was collected and dispensed into several sterile 50 mL centrifuge tubes (SPL Lifesciences, South Korea). The untreated samples were transported directly to the laboratory within 6 h after collection and stored at 4°C.

### Total DNA extraction

Total fecal DNA was extracted using the manufacturer’s protocol of the QIAamp DNA Feces mini kit (Qiagen, Germany) with two modifications of the optimized lysis buffer (500 mM NaCl, 50 mM Tris–HCl (pH 8.0), 50 mM EDTA (pH 8.0), and 4% sodium dodecyl sulfate) from the previous paper (Ku and Lee, 2014) and an additional 10 min boiling step. After homogenization of 0.25 g feces with 1 mL optimized lysis buffer, the fecal suspension was incubated for 10 min in boiling water to lyse the cells. In the final step, fecal DNA was eluted using 100 μL of molecular water (Welgene, South Korea). The extracted total fecal DNA was quantified using NanoDrop 2000 (Thermo Scientific, United States). For extraction of total bacteriophage DNA from collected sewage samples, sewage components and bacteria were removed by centrifugation at 11,000 × g for 30 min and subsequent filtration with Acrodisc syringe filters (Pall, United States; pore size = 0.45 μm). After their removal, the filtrate solutions (final volume, 10 mL) were used for total bacteriophage DNA extraction using the manufacturer’s protocol of the QIAamp DNA Blood Maxi Kit (Qiagen). After the food application tests, total bacteriophage DNA was extracted from homogenized food samples using the Viral Gene-spin Viral DNA/RNA Extraction Kit (Intron Biotechnology, South Korea) following the standard manual procedure.

### Conventional PCR

All conventional PCRs were performed using a C1000 Touch Thermal cycler (Bio-Rad, United States). The reaction mixture (final volume, 25 μL) was prepared with 100 ng of template DNA, 15 μM of each forward and reverse primer set, 0.2 mM of each dNTP, 1× *Taq* PCR buffer, and 0.5 unit of *Taq* DNA polymerase (MGmed, South Korea). The PCR reaction condition was as follows: an initial denaturation step at 95°C for 3 min, followed by 35 cycles of 95°C for 10s, 50°C for 30s, and 72°C for 1 min, and a final extension step at 72°C for 5 min. After PCR amplification, PCR products were purified using the AxyPrep DNA Gel Extraction Kit (Axygen, United States) and quantified using NanoDrop 2000 (Thermo Scientific). To confirm the PCR products, agarose gel electrophoresis was performed on a 2.5% Molecular Biology Certified Agarose gel (Bio-Rad) and visualized using a Gel Doc EZ Imager (Bio-Rad).

### Quantitative real-time PCR

For qPCR, a TaqMan real-time PCR assay was conducted with a specifically optimized qPCR reaction mixture for each TaqMan primer/probe combination using the Bio-Rad CFX connect Real-Time System (Supplementary Table S1). The qPCR reaction condition to detect crAssphage DNA is as follows: predenaturation at 95°C (10 min), followed by denaturation at 95°C (10 s) for 39 cycles, and annealing at 60°C (30 s) for 39 cycles. To confirm DNA amplification, amplified qPCR products were observed on a 2.5% Molecular Biology Certified Agarose gel (Bio-Rad) and visualized using a Gel Doc EZ Imager (Bio-Rad).

### Sensitivity test and standard curve

To determine the detection limit of each primer/probe combination in the qPCR assay, full-length amplicons of ORF00018, ORF00024, and ORF00044 were obtained from a crAssphage-positive human fecal sample under conventional PCR conditions with specific combinations (Table 1). Subsequently, these amplicons were quantified with NanoDrop 2000 (Thermo Scientific) after gel extraction using the AxyPrep DNA Gel Extraction Kit (Axygen). The qPCR template DNA samples were prepared with a 10-fold serial dilution from 0.1 fg/μL to 1 ng/μL. With these diluted template DNAs, qPCR was performed with each primer/probe combination, and its detection limit was determined with the lowest concentration of template DNA for which the amplification signal was detected. The standard curve of each combination was determined with a correlation between the template DNA concentrations and the C_q_ values, and its accuracy was confirmed with *R^2^* exceeding 0.95.

### Quantification of crAssphage

To quantify the viral load of crAssphage in human fecal samples or untreated sewage samples, the template DNA concentration in the qPCR reaction was calculated using the C_q_ value with the equation obtained from the standard curve. The value of DNA concentration was converted from “ng/g of feces” to “log_10_ (genomic copies/g of feces or mL of sewage)” with the following equation:


$$\text{Number of copies}=\frac{\text{Amount of}\;\text{DNA}\;\left(\text{ng}\right)\times 6.022\times 10^{23}\;\left(g/\text{mol}\right)}{\text{Length}\;\left(\text{bp}\right)\times 1\times 10^{9}\;\left(\text{ng}/g\right)\times 660\;\left(g/\text{mol}\;\text{of}\;1\;\text{bp}\;\text{dsDNA}\right)}$$

### Food application

To detect human fecal contamination of foods, fresh romaine lettuce and ground beef samples were collected from local grocery markets (Yongin, Korea), and 1, 0.1, 0.01, and 0.001 g of the selected crAssphage-positive human fecal sample (AD8, 10 ^10.55^ copy numbers/g stool sample) were inoculated onto 1 g of each food sample. After 1 h drying at room temperature, each mixture of food and human feces was resuspended and adjusted to 10 mL (final volume) with sterilized 0.1% peptone water, after which the resuspended solution was placed in a 3 M Plain Sample Bag and homogenized for 30 s using the BagMixer 400 CC stomacher (Intersciences, United States). After stomaching, the homogenized solution was transferred to sterile 15 mL centrifuge tubes (SPL Lifesciences) and centrifuged at 11,000 × g for 10 min. After centrifugation, the supernatant was collected and used for total DNA extraction. The extracted total DNA was used as a template for qPCR detection of crAssphage.

### Statistical analysis

Pearson’s Chi-squared test for the positive rates of crAssphage between different health condition groups was conducted with the SPSS program (ver. 25). Differences were defined as significant at *p* < 0.05.

### Institutional review board (IRB) approval

This study was approved by the IRB of Kyung Hee University (South Korea) to obtain human fecal samples. The approval number is KHGIRB-19-192.

## Results

### Design of oligonucleotide primer/probe combinations

The sequences of the uncultured crAssphage complete genome (accession no. JQ995537.1) and uncultured phage crAssphage clone ICD-206 polymerase (P) gene (accession no. KX342816.1) were obtained from the GenBank database.^1^ CrAssPFL1 primer/probe combination targeting ORF00018 (putative DNA polymerase) of uncultured crAssphage complete genome was designed using the GenScript Real-Time PCR primer design program to produce a PCR product at the range of 100 to 200 bp with a melting temperature (T_m_) of 50°C–55°C. The CrAssPFL2 primer/probe combination targeting ORF00044 (hypothetical protein) was also designed using the program with the same parameters. Other crAssphage primer/probe combinations, CrAssBP (193 bp) targeting ORF00018, CPQ056 (126 bp) targeting ORF00024, and RQ (182 bp) targeting ORF00044, were obtained from previous reports (Stachler et al., 2017; Cinek et al., 2018; Park et al., 2020). The predicted sizes of the conventional PCR products with CrAssPFL1 and CrAssPFL2 were 200 and 197 bp, respectively. Table 1 lists the sequences of all primer/probe combinations.

### Evaluation of PCR product size and primer specificity by conventional PCR

**Figure 1:**
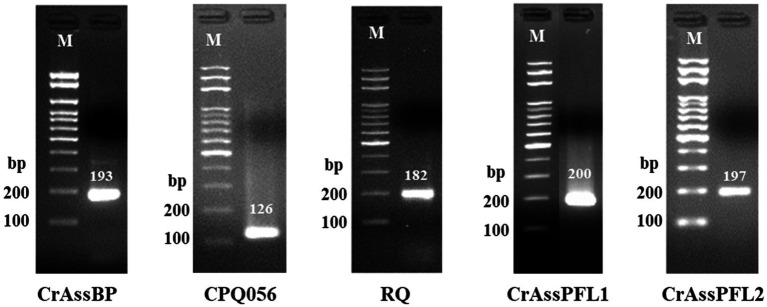
Results of conventional PCR. The results of agarose gel electrophoresis showed PCR amplification of DNA extracted from a South Korean adult fecal sample. Each oligonucleotide primer/probe combination that targets different regions of crAssphage on 2.5% agarose gel forms one specific band. Lane M indicates the MG 100 bp DNA ladder marker (MGmed, South Korea). The second lane of each gel is the result of PCR of DNA extracted from Korean adult fecal samples.

To evaluate the PCR product size and specificity of each combination, the human fecal DNA samples were selected and amplified using the conventional PCR method with CrAssPFL1, CrAssPFL2, CrAssBP (Park et al., 2020), CPQ056 (Stachler et al., 2017), and RQ (Cinek et al., 2018). After PCR amplifications, subsequent agarose gel electrophoresis showed that the PCR product sizes exactly matched and were confirmed to the predicted or reported sizes: 193 bp for CrAssBP, 126 bp for CPQ056, 182 bp for RQ, 200 bp for CrAssPFL1, and 197 bp for CrAssPFL2, respectively (Figure 1). In addition, all PCR products were only single-specific bands present in the agarose gel, suggesting that all combinations amplified specifically only target sites on the crAssphage genome (Figure 1).

### Composition optimization of qPCR mixture

To optimize the composition of the qPCR mixture for each primer/probe combination, previously reported optimum qPCR compositions of CrAssBP and RQ were referenced and the ranges of qPCR components were determined. As the optimum qPCR composition of CPQ056 was not reported, the qPCR compositions of CPQ056 were also optimized with CrAssPFL1, and CrAssPFL2. Before optimization, 10 different qPCR compositions were determined and tested with the ranges of F/R primers (0.55 to 1.2 μM) and TaqMan probe (0.16 to 0.6 μM) (Supplementary Table S1). The optimal qPCR compositions of CrAssBP and RQ were confirmed to be conditions 10 and 4, which are consistent with previously published compositions (Supplementary Figures S1A,S1C). In addition, the optimal qPCR compositions of CPQ056, CrAssPFL1, and CrASSPFL2 were conditions 1, 2, and 3, respectively (Supplementary Figures S1B,S1D,S1E). Therefore, based on these results, the final optimal qPCR compositions are summarized in Table 2 and used for further qPCR reactions in this study.

**Table 2:** Optimization of primer/probe combinations composition for qPCR.

Sample	CrAssBP	CPQ056	RQ	CrAssPFL1	CrAssPFL2
Template DNA (ng)	10	5	10	5	5
F primer (20 μM) (μL)	1.5	0.68	0.75	0.75	0.75
R primer (20 μM) (μL)	1.5	0.68	0.75	0.75	0.75
TaqMan probe (20 μM) (μL)	0.5	0.68	0.2	0.75	0.3
qPCR mix (2X) (μL)	12.5	12.5	12.5	12.5	12.5
ROX Reference dye II (μL)	0.4	0.4	0.4	0.4	0.4
Molecular water (μL)	6.6	9.06	8.4	8.85	9.3
Total volume (μL)	25	25	25	25	25
Reference	Liang et al. (2016)	In this study	Stachler et al. (2017)	In this study	In this study

### Evaluation of crAssphage detection by optimized qPCR

**Figure 2:**
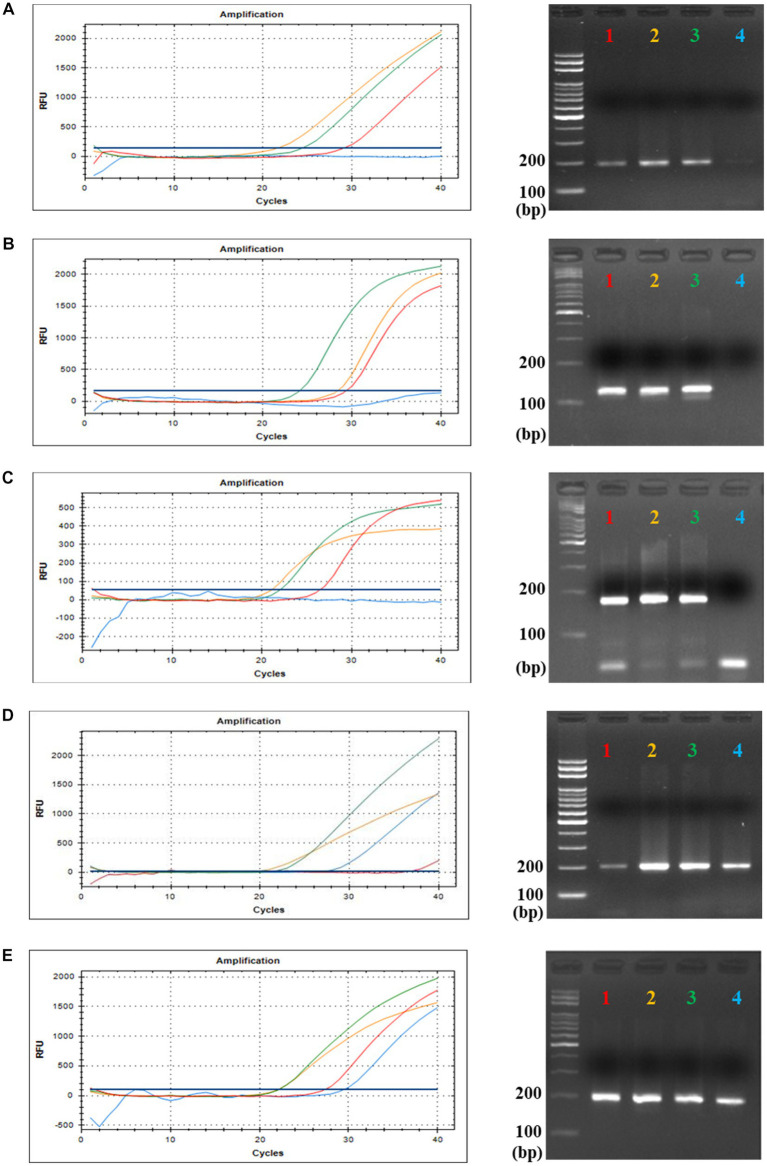
qPCR and gel electrophoresis results of the four Korean fecal samples. This is the result of TaqMan Real-Time PCR with each TaqMan probe targeting five specific sequences of crAssphage; **(A)** CrAssBP targeting ORF00018 of crAssphage (193 bp). **(B)** CPQ056 targeting ORF00024 of crAssphage (126 bp). **(C)** RQ targeting ORF00044 of crAssphage (182 bp). **(D)** CrAssPFL1 targeting ORF00018 of crAssphage (200 bp). **(E)** CrAssPFL2 targeting ORF00044 of crAssphage (196 bp). Lane 1 (Red), 11-year-old male child’s fecal sample; Lane 2 (Orange), 14-year-old male adolescents’ fecal sample; Lane 3 (Green), 12-year-old male adolescents’ fecal sample; Lane 4 (Blue), 15-year-old male adolescents’ fecal sample.

To confirm the optimized mixture composition and to evaluate the specificity of each primers/probe combination, four different crAssphage-positive fecal DNA samples were selected (Sample 1, 11-year-old male; Sample 2, 14-year-old male; Sample 3, 12-year-old male; Sample 4, 15-year-old male) and used for this qPCR evaluation test. All qPCR products of each primer/probe combination were only one single band in gel electrophoresis, and the product sizes exactly matched the predicted ones, suggesting specific detection of crAssphage in human fecal samples (Supplementary Table S2 and Figure 2). Interestingly, while crAssphage in human fecal Samples 1, 2, and 3 was detected with all primer/probe combinations, that of Sample 4 was detected only with CrAssPFL1 and CrAssPFL2,

### Verification of human specificity of crAssphage

**Figure 3:**
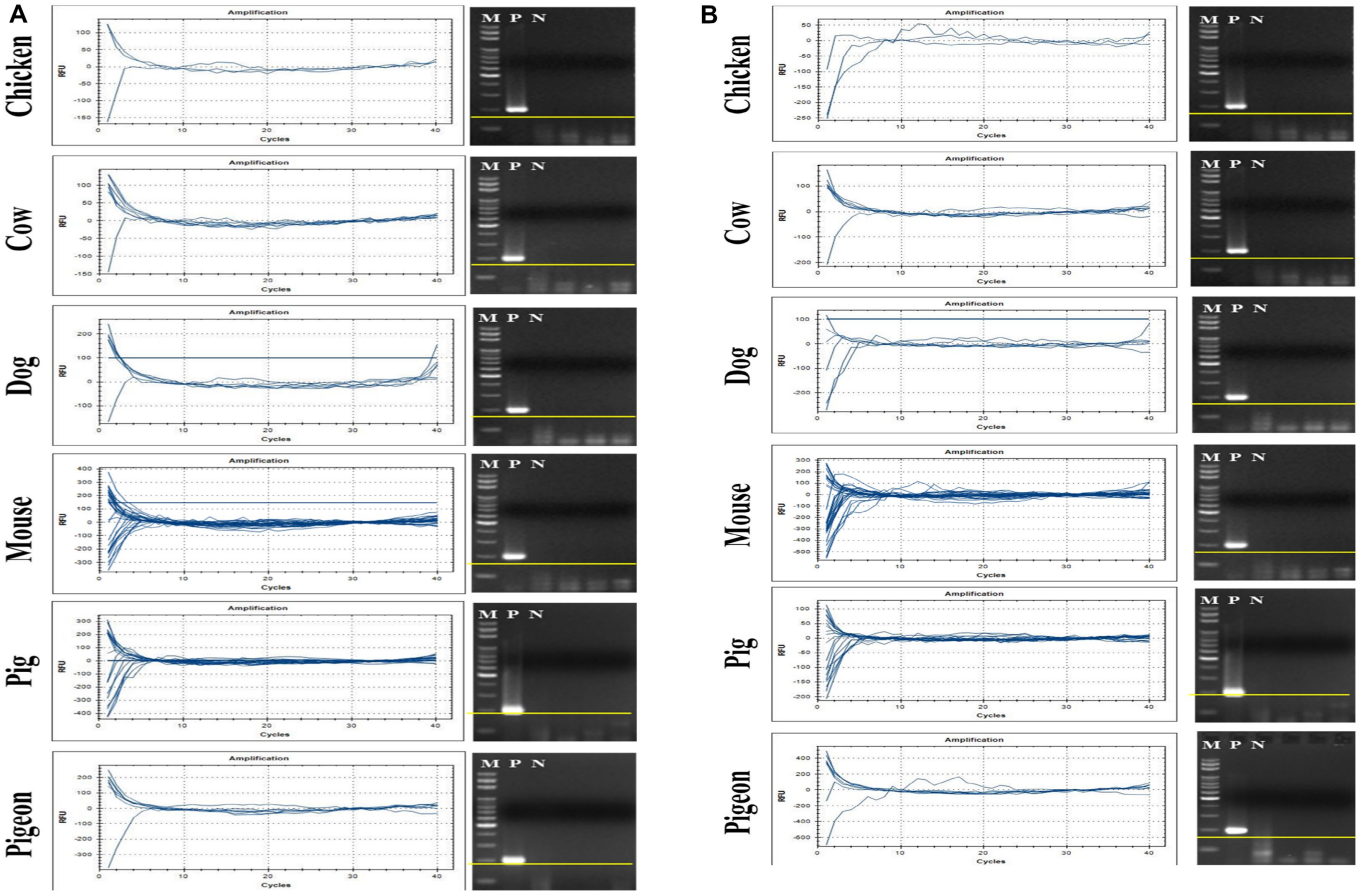
qPCR results for CrAssPFL1 and CrAssPFL2. qPCR with the primer/probe combination **(A)** CrAssPFL1 or **(B)** CrAssPFL2 was conducted to amplify DNA extracted from the feces of each animal. Lanes M, 100 bp DNA ladder marker (MGmed); Lanes P, human fecal DNA; Lanes N, molecular water.

Eighty-nine fecal samples from six different animals were collected, and their extracted total fecal DNAs were used as template DNA for conventional PCR and qPCR reactions with crAssphage-specific primer/probe combinations. As positive controls, a crAssphage-positive human fecal sample was randomly selected, and its conventional PCR with the CrAssPFL1 or CrAssPFL2 was performed, consistently showing a single PCR amplicon band (200 bp) (Figure 2). On the other hand, subsequent conventional PCR reactions in triplicate with a fecal DNA mixture of each animal as template DNA and the same combination showed no specific PCR amplicon band in the agarose gel. To further verify the human specificity of crAssphage, a qPCR reaction was conducted with each fecal DNA sample from 89 animal fecal samples as a template DNA. As expected, the qPCR reactions with the CrAssPFL1 or CrAssPFL2 showed no amplification signal in the results (Figures 3). Furthermore, these conventional PCR and qPCR with the other three crAssphage-targeting primer/probe combinations (CrAssBP, CPQ056, and RQ) also showed no PCR amplicon or qPCR signal (Supplementary Figure S2). Overall, these results confirm that conventional PCR and even qPCR with five different crAssphage-targeting primer/probe combinations can detect crAssphage only in human fecal samples.

### Distribution and prevalence of crAssphage in human fecal samples

So far, only one study has reported crAssphage distribution in South Korea using qPCR with CPQ064, showing a 39% detection rate (37 positive out of 94 fecal samples). To further understand crAssphage distribution and prevalence in South Korea, 139 Korean fecal samples containing 37 healthy people and even 102 gut-associated patients (35 IBS patients and 67 CRC patients) were collected, and the qPCR detection of crAssphage in these samples was performed with five different crAssphage-targeting primer/probe combinations (crAssBP, CPQ056, RQ, CrAssPFL1, and CrAssPFL2). The crAssphage detection rates of these five combinations were 28.8, 30.2, 23.0, 20.1, and 30.9%, respectively.

### Correlation between crAssphage detection ratio and health status

To evaluate the possibility of association between the existence of crAssphage and human health status, the detection rates of crAssphage between 37 healthy South Korean people and 102 gut-associated patients (35 IBS patients, and 67 CRC patients) were compared. The qPCR detection of crAssphage in South Korea with five different crAssphage-targeting primer/probe combinations revealed that the range of detection rates among all 139 fecal samples was 20.1–30.9% (Table 3). The qPCR detection rate of crAssphage in fecal samples with the CrAssPFL2 was 29.7% (12/37) of healthy adults, 34.3% (12/35) of IBS patients, and 29.9% (20/67) of CRC patients (Table 3). To verify the association between crAssphage and gut-associated diseases, Pearson–Chi Square data analysis using the qPCR detection results of 139 fecal samples with a CrAssPFL2 was conducted. However, this statistical analysis confirmed that there is no significant correlation between the ratio of crAssphage-positive subjects and health status (*p* > 0.05, Pearson’s Chi-squared test) (Table 4), suggesting that the presence of crAssphage in the gut is not associated with gut-associated disease.

**Table 3:** Detection efficiency of each primer/probe combination according to the health status of Koreans.

	CrAssBP	CPQ056	RQ	CrAssPFL1	CrAssPFL2
Healthy	32.4% (12/37)	32.4% (12/37)	24.3% (9/37)	16.2% (6/37)	29.7% (11/37)
Irritable Bowel Syndrome	25.7% (9/35)	28.6% (10/35)	17.1% (6/35)	28.6% (10/35)	34.3% (12/35)
Colorectal cancer	28.3% (19/67)	29.8% (20/67)	25.4% (17/67)	17.9% (12/67)	29.9% (20/67)
Total	28.8% (40/139)	30.2% (42/139)	23% (32/139)	20.1% (28/139)	30.9% (43/139)

**Table 4:** Pearson–Chi Square data analysis.

Pearson–Chi Square			Detection		x^2^ ^a^/*p*
			Positive	Negative	
Health status (*n* = 139)	Healthy (*n* = 37)	Count	11	26	0.246/0.884
		Expected Count	11.4	25.6	
	Irritable bowel syndrome (*n* = 35)	Count	12	23	
		Expected Count	10.8	24.2	
	Colorectal cancer (*n* = 67)	Count	20	47	
		Expected Count	20.7	46.3	

a X2 = ∑ (count–expected count)2/expected count.

### Sensitivity test

**Figure 4:**
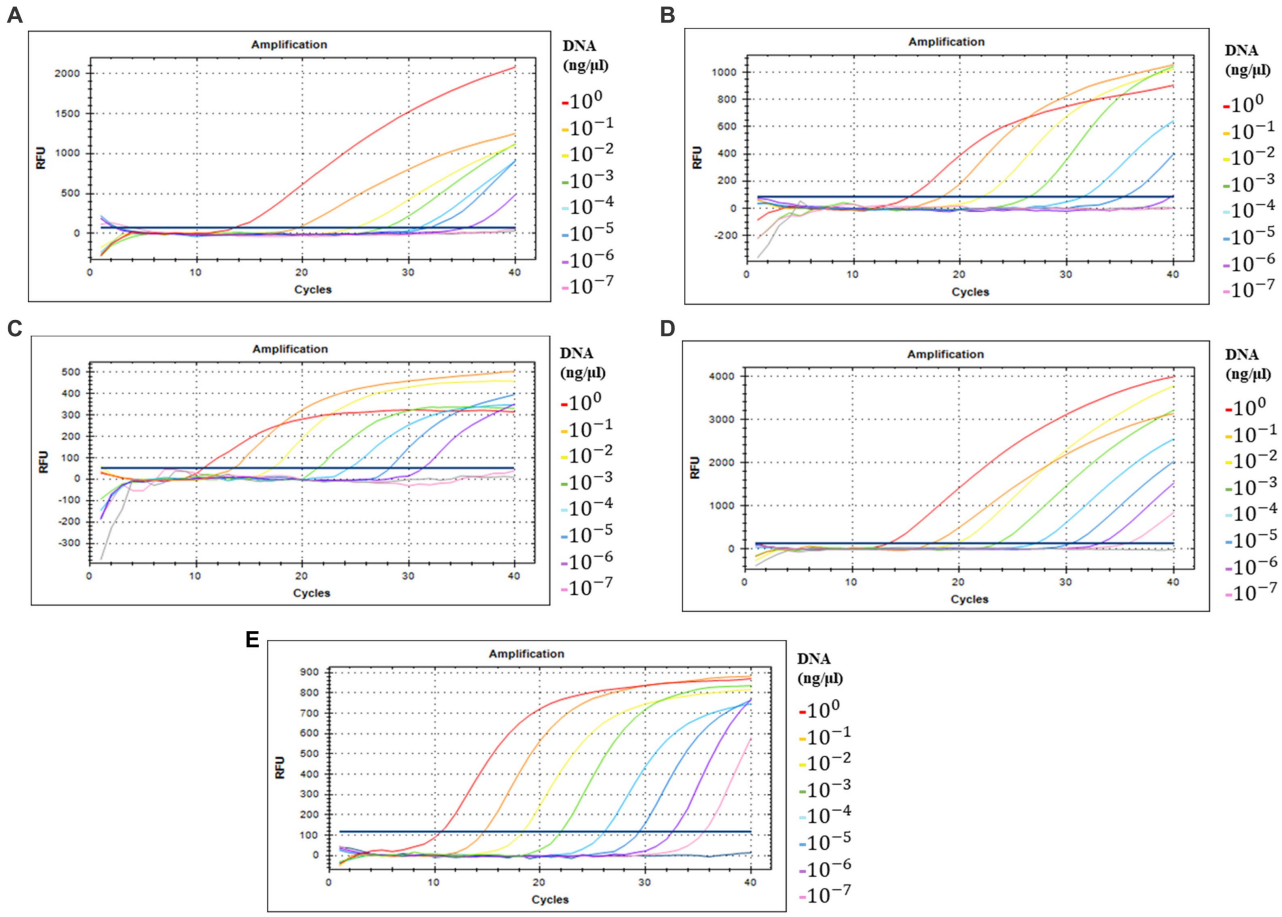
Amplification curves by qPCR using each primer/probe combination; **(A)** CrAssBP **(B)** CPQ056 **(C)** RQ **(D)** CrAssPFL1 **(E)** CrAssPFL2. Templates were the products of the crAssphage genome by conventional PCR. Each gene in crAssphage was diluted 1/10 in 8 steps.

Detection sensitivity analysis of five primer/probe combinations in qPCR with serially diluted DNA concentrations of full-length amplicons of ORF00018 (CrAssBP and CrAssPFL1), ORF00024 (CPQ056), and ORF00044 (RQ and CrAssPFL2) as DNA templates was performed to determine the detection limit (Supplementary Table S3 and Figure 4). This analysis revealed that crAssphage could be detected at concentrations ranging from 1 to 10 fg/μL of the amplicons. Interestingly, while the detection limit of crAssphage using RQ was 1 fg/μL, that of CrAssPFL2 was 0.1 fg/μL, suggesting that the CrAssPFL2 combination has higher sensitivity to crAssphage, which targets the same gene, ORF00044 (Figures 4C,E). In addition, the comparison of the detection limits between CrAssBP and CrAssPFL1 targeting the same gene (ORF00018) showed the detection limit of CrAssPFL1 was higher (0.1 fg/μL) than the detection limit of CrAssBP, 1 fg/μL (Figures 4A,D). Furthermore, the detection limit of crAssphage using CPQ056 was 1 fg/μL (Figure 4B).

### Quantification of crAssphage in human fecal and sewage samples

**Figure 5:**
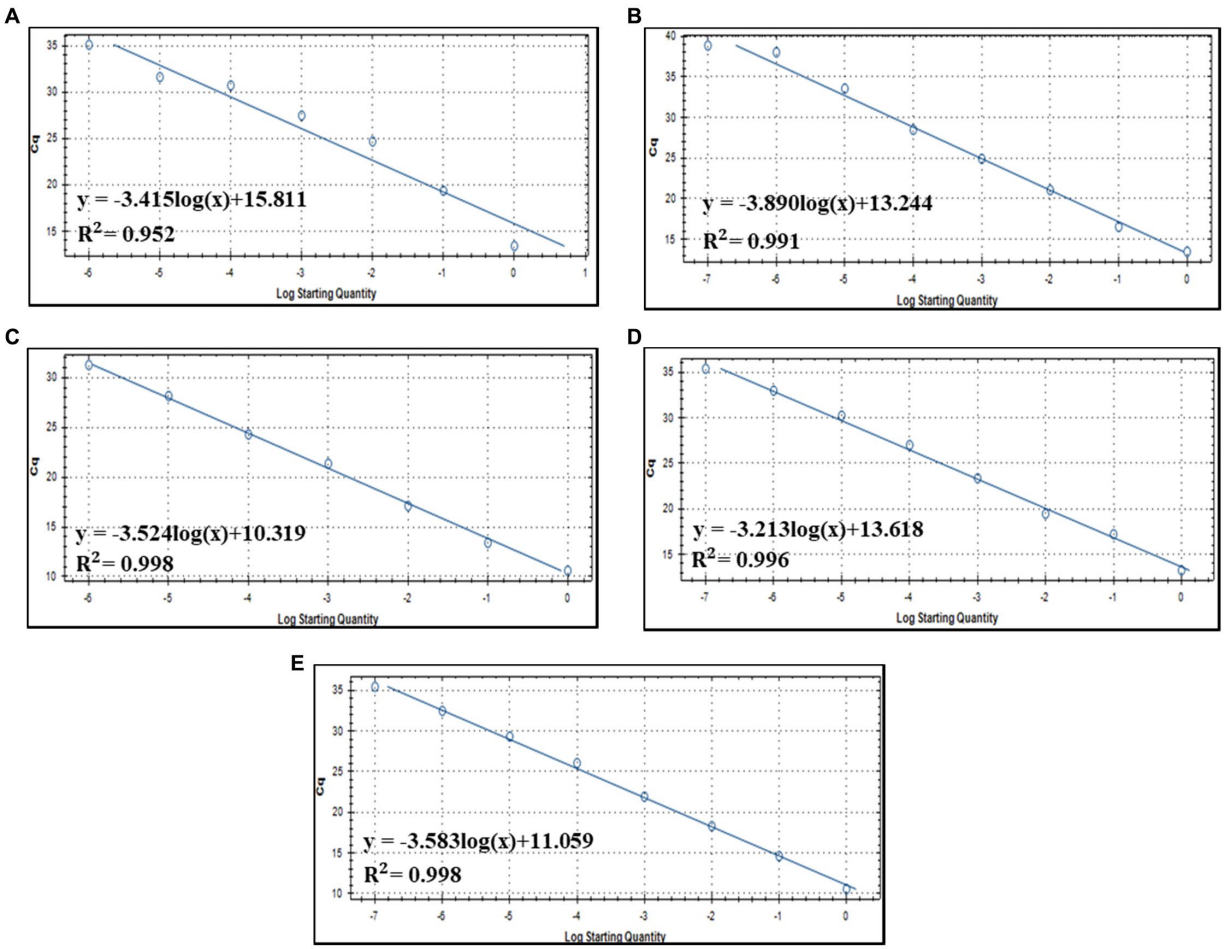
Standard curve of qPCR detection assay using each primer/probe combination; **(A)** CrAssBP **(B)** CPQ056 **(C)** RQ **(D)** CrAssPFL1 **(E)** CrAssPFL2.

Notably, the change in Cq values was consistent with the concentrations of serially diluted fecal DNAs, suggesting the possibility of quantifying crAssphage using qPCR. Therefore, the standard curves between the Cq value of crAssphage detection and the related specific DNA concentration of crAssphage amplicons were drawn (Figure 5). The correlation coefficient (*R^2^*) between the Cq value and amplicon concentration was 0.952, 0.996, 0.991, 0.998, and 0.998 for CrAssBP (Figure 5A), CPQ056 (Figure 5B), RQ (Figure 5C), CrAssPFL1 (Figure 5D), and CrAssPFL2 (Figure 5E), respectively. Based on this statistical result, with a coefficient value of CrAssPFL2 (0.998), the qPCR assay using this combination could be the most accurate for the quantification of crAssphage in fecal samples.

**Figure 6:**
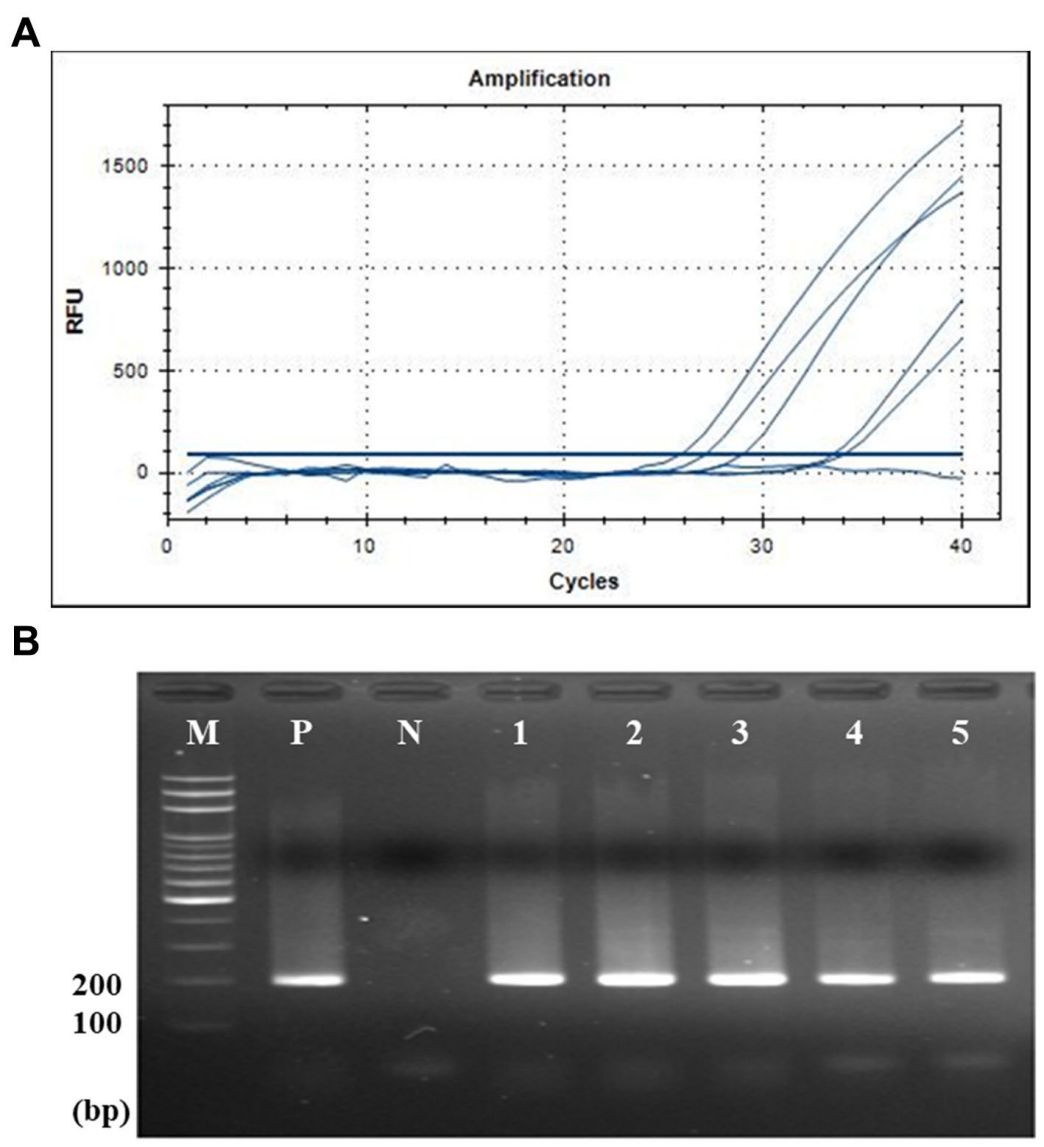
qPCR and gel electrophoresis results of five sewage samples. **(A)** qPCR with CrAssPFL2. Molecular water was used as a negative control. The Ct value of crAssphage detected in sewage samples is 20–34 cycles. **(B)** Gel electrophoresis after qPCR. Lanes M, 100 bp DNA ladder marker (MGmed); Lanes P, human fecal DNA; Lanes N, molecular water; Lanes 1, Opo sewage treatment plant (STP); Lanes 2, Gyongan STP; Lanes 3, Seongnam Water Quality Restoration Center; Lanes 4, Bongdam STP; Lanes 5, Gwangju STP.

According to the standard curve between the Cq value and amplicon concentration, the quantification of seven human fecal samples was conducted. The amount of crAssphage was quantified as viral load, the log value of the crAssphage genomic copies per gram of feces [log_10_ (genomic copies/g feces)] using each primer/probe combination. This quantification assay showed that the CrAssPFL2 primer/probe combination can detect average 10^9.43^ of copies per gram of feces, which was the highest viral load among the five primer/probe combination (10^9.01^, 10^9.11^, 10^8.67^, and 10^9.17^ of copies per gram of feces using CrAssBP, CPQ056, RQ, and CrAssPFL1, respectively) (Table 5). This result supports that the CrAssPFL2 combination is more sensitive than other ones. Therefore, this combination was selected to quantify the viral load of crAssphage in the collected sewage samples. The qPCR assay using CrAssPFL2 showed that all five sewage samples had crAssphage (Figure 6), and the Cq value of each sewage sample was determined (data not shown). The quantification assay with the Cq values revealed that crAssphage in the sewage samples ranged from 10^5.23^ to 10^6.38^ of copies per mL of sewage, indicating 3–4 log lower than the value detected from human feces (Table 6).

**Table 5:** Quantification of crAssphage in Korean fecal samples.

Korean fecal samples^a^	CrAssBP	CPQ056	RQ	CrAssPFL1	CrAssPFL2
AS14	7.82	8.81	6.94	8.36	8.36
AS15	9.16	8.58	9.23	9.89	10.00
AS21	8.74	7.91	8.43	8.32	7.92
AS27	9.14	9.67	8.96	9.27	9.19
AS30	9.71	10.52	9.51	9.66	10.47
AD6	8.89	8.66	8.85	9.03	9.56
AD8	9.59	9.63	8.79	9.65	10.55
Average	9.01	9.11	8.67	9.17	9.43

^a^The quantification value was shown as viral load, the value of the log on the crAssphage genomic copies per g of feces [log_10_ (genomic copies/g feces)]. AS, adolescent with IBS; AD, healthy adult.

**Table 6:** Quantification of crAssphage in sewage samples using CrAssPFL2.

No.	Sewage samples	Viral load^a^
1	Opo Sewage Treatment Plant	6.38
2	Gyeongan Sewage Treatment Plant	5.23
3	Seongnam Water Quality Restoration Center	5.98
4	Bongdam Sewage Treatment Plant	5.46
5	Gwangju Sewage Treatment Plant	5.5

a Viral load: log_10_ (genomic copies/mL of sewage).

### Food application

**Figure 7:**
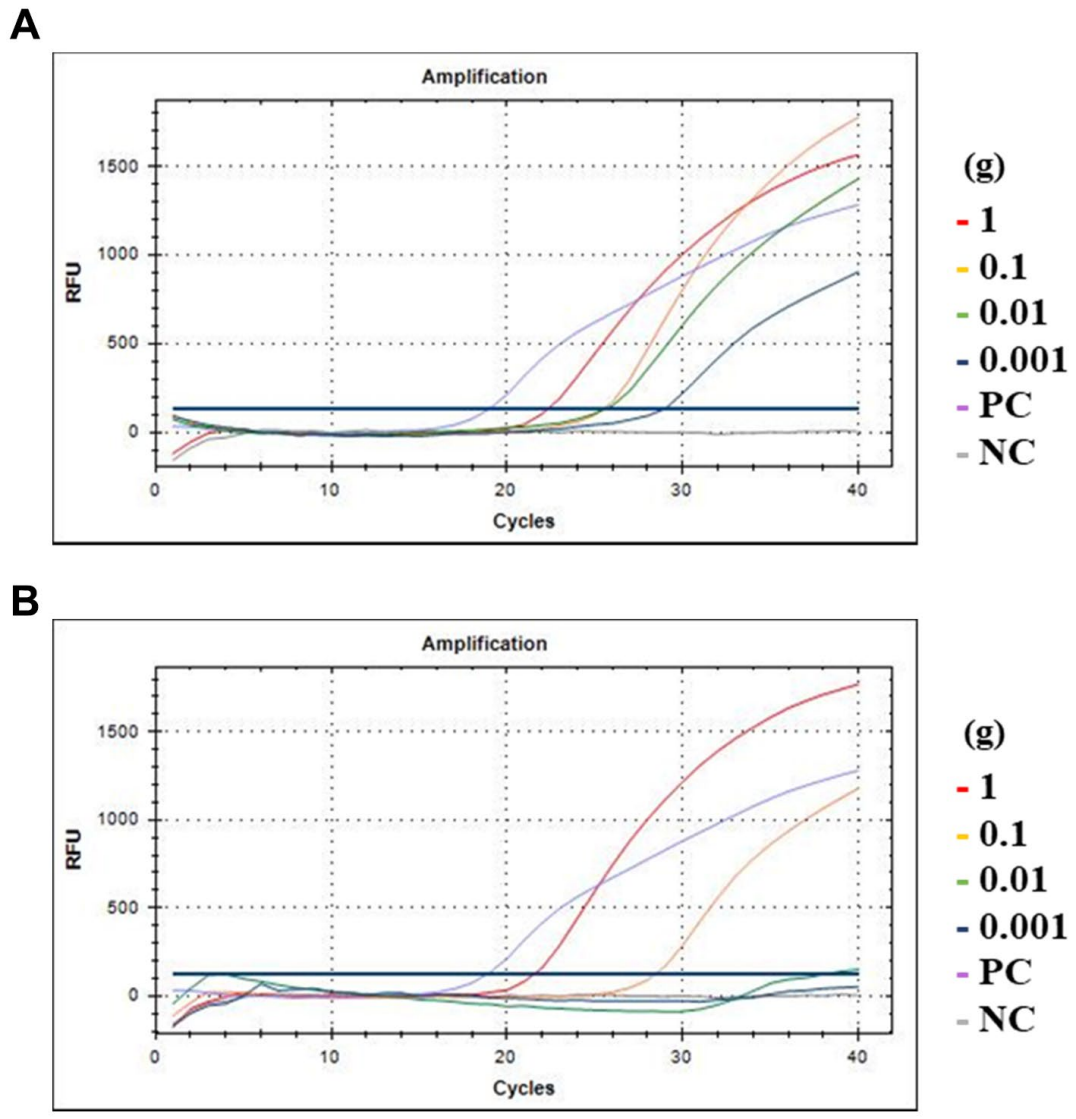
Amplification curves of qPCR assay with food samples. **(A)** Romaine lettuce; **(B)** Ground beef. The red, yellow, green, and blue lines indicate 1, 0.1, 0.01, and 0.001 g of human feces, respectively. PC at purple line, DNA of crAssphage isolated from Gwang-ju sewage treatment plant as positive control; NC at gray line, the same amount of molecular water as a negative control.

To evaluate the detection efficiency of crAssphage using the CrAssPFL2 primer/probe combination in food environments, fresh romaine lettuce and ground beef were artificially contaminated with the crAssphage-positive human fecal sample, AD8 (10 ^10.55^ copy numbers/g stool sample). In the contaminated romaine lettuce, crAssphage was detected up to 0.001 g of the selected fecal sample (10^7.55^ copy numbers). However, in ground beef, it was detected up to 0.1 g of the same fecal sample (10^9.55^ copy numbers), suggesting that PCR inhibitors, such as fat, protein, and collagen, generally present in beef samples could affect this low detection (Figure 7).

## Discussion

Human-specific crAssphage is considered the best indicator to evaluate human fecal contamination in environments and even foods (Ahmed et al., 2018, 2019; Kongprajug et al., 2019). So far, several qPCR oligonucleotide primer/probe combinations, including CrAssBP (Park et al., 2020), CPQ056 (Stachler et al., 2017), and RQ (Cinek et al., 2018), have been developed and reported. The abundance of crAssphage detected by CPQ056 varied from 38 to 71% (Stachler et al., 2017; Ahmed et al., 2018). RQ, which targets ORF00042 (hypothetical protein), had a detection rate ranging from 10.7 to 31.4%, depending on the country of origin (Cinek et al., 2018). Another combination, CrAssBP, targeting ORF0018 (DNA polymerase), detected crAssphage in 48–68.5% of fecal samples from healthy subject and in 71.4% of norovirus-positive human fecal samples (Park et al., 2020). However, it is still necessary to develop and optimize a new crAssphage-targeting qPCR primer/probe combination with enhanced detection efficiency and sensitivity. In this study, two new crAssphage-targeting qPCR primer/probe combinations were developed, and demonstrated that the CrAssPFL2 outperformed others for human specificity, and sensitivity.

To evaluate the performance of the qPCR detection method in this study, the qPCR efficiency should be considered with the standard curve of each primer/probe combination, referring to a previous study (Peirson et al., 2003). qPCR efficiency is defined as the fraction of DNA amplified in one PCR reaction (Svec et al., 2015). It is needed to determine the assay dynamic range, limit of detection, and limit of quantification when designing a new primer/probe combination in qPCR assay, because this value depends on (1) various parameters such as primers’ and template sequences and structures, (2) reagents used for dilution, and (3) sample matrix. The qPCR efficiencies of CrAssPFL1 and CrAssPFL2 were 105 and 90%, respectively. In general, several other studies have considered values between 90 and 110% acceptable (Robledo et al., 2014; Sultana et al., 2020). Therefore, qPCR detections with these new combinations would be reliable. At the same time, the qPCR efficiencies of CrAssBP, CPQ56, and RQ in this study were 96, 81, and 92%, respectively, suggesting that the CrAssBP and RQ primer/probe combinations remain useful for detecting crAssphage. Based on this comparative result, the combination CrAssPFL2 had the highest detection performance in this study. The highest number of crAssphage detection with CrAssPFL2 (43 of 139 fecal samples, 30.9%; See Table 3) support this.

Considering the results of the crAssphage distribution and prevalence in this study, the detection rate of crAssphage was 20.1–30.9%. According to previous studies that explored crAssphage identification and prevalence, a trend exists between the detection rates of Western and Eastern human fecal samples. The detection rates in Western regions such as Australia (Ahmed et al., 2018), the United States (Park et al., 2020), and Spain (García-Aljaro et al., 2017) are relatively higher than that in Eastern regions such as South Korea (Nam et al., 2022), China (Liang et al., 2018; Li et al., 2021), Nepal (Ward et al., 2020), and Thailand (Kongprajug et al., 2019). For instance, in a detection experiment using the combination CrAssBP, crAssphage was detected in 45 of 68 American fecal samples (66.2%) (Park et al., 2020). In addition, in the detection experiment using the combination CPQ056 from 13 Australian feces, crAssphage was detected in 6 of 13 fecal samples (46.1%) (Ahmed et al., 2018). However, the detection rate of the Asian feces was distributed at a relatively low level. For the detection ratio of crAssphage in Chinese fecal samples, 49 of 255 samples (19.2%) had crAssphage in the samples when primers targeting ORF00018 like CrAssBP were used (Liang et al., 2018), whereas another report detected crAssphage in 124 of 256 fecal samples (48.4%) in Chinese human gut samples (Chen et al., 2021). The crAssphage detection rate in South Korea using CPQ064 was recently reported to be 39% (37 of 94 fecal samples) (Nam et al., 2022), similar to this study. So far, only two strains of crAssphage specific to *Bacteroides* have been isolated and characterized, suggesting that its host is assumed to be *Bacteroides* sp. (Dutilh et al., 2014). Therefore, crAssphage abundance may be associated with the dominance of *Bacteriodes* in the gut habitat. Notably, a number of gut microbiome studies have suggested that *Bacteroides* sp. is predominant in the gut of people who consume a high-protein/low-carbohydrate Western diet, whereas *Prevotella* sp. mainly colonizes in the gut of people who consume a high-carbohydrate/low-protein Eastern diet (Gorvitovskaia et al., 2016; Ahmed et al., 2018). The genus *Bacteroides* is known to be affected by the host diet. For instance, a previous study reported that long-term consumption of animal foods might be positively correlated with the prevalence of *Bacteroides* in the gut (Tomova et al., 2019). In addition, fecal bile acid concentration has been reported to be associated with animal-based dietary patterns because bile acid plays an important role in animal fat metabolism (Trefflich et al., 2019). This result suggests why *Bacteroides* is predominant in the gut microbiota of Western people consuming animal-based foods because *Bacteroides* is known to have bile tolerance activity for survival in the gut environment (Tomova et al., 2019). However, further research is needed to explain high prevlance of crAssphage in Western people over Asians.

Although crAssphage exists across various regions and races, the correlation between crAssphage in the human gut and intestinal diseases has not been properly clarified (Honap et al., 2020). In this study, the crAssphage-positive rate over intestinal disease did not show significance in Pearson’s chi-squared analysis, suggesting that further study may be needed to investigate if crAssphage abundance is associated with intestinal diseases. According to previous studies, the intestinal microbiome, especially the abundance of *Bacteroides*, is affected by intestinal diseases, such as IBS, Crohn’s disease, and CRC (Wang et al., 2021; Zafar and Saier, 2021). Moreover, a recent study reported that crAssphage subfamilies showed different stability in the human phageome dependent on the abundance of *Bacteroidales* in the gut of people suffering from obesity and metabolic syndrome (Cervantes-Echeverria et al., 2022), suggesting that the association between human diseases and crAssphage prevalence may be dependent on the abudance of host species of each crAssphage subfamily. However, those crAssphage-targeting primer/probe combinations cannot discriminate its subfamilies for detection and monitoring at this time. Therefore, the response and change of this crAssphage subfamily level is undetectable. To overcome these limitations of crAssphage primer/probe combinations, it is necessary to further study the nature of crAssphage to extend its scientific information to the subfamily level. This information would be useful for developing the new crAssphage subfamily-specific primer/probe combinations with more accurate and delicate detection markers in the subfamilies.

Although the presence of crAssphage was discovered first in human fecal samples (Dutilh et al., 2014) and the crAssphage isolates ΦcrAss001 and ΦcrAss002 were also isolated from human fecal samples (Shkoporov et al., 2018; Guerin et al., 2021), *Bacteroides* is also prevalent in animal gut microbiota, implying on the possible presence of crAssphage in the animal gut microbiota. However, no report for crAssphage detection was announced in animal fecal samples. The most recently, a few qPCR analyses targeting human crAssphage revealed that the crAssphage may be present in the animal gut microbiota humans (Ahmed et al., 2018, 2019). However, these animals are strongly related to humans, because they are all companion animals and feedstock animals. Actually, these crAssphage-positive animals shared their living space with humans in that studies and their detection rates were not consistent as well as lower than the rates in humans. Therefore, it is thought that these crAssphage may be transmitted from humans to the closely related animals. To further understand this association between humans and animals for transmission of crAssphage, extensive experiments need to be done.

CrAssphage is one of the best indicators to confirm human fecal contamination in environments as well as foods. In this study, two new crAssphage-targeting primer/probe combinations and three previously reported combinations were evaluated, and their qPCR conditions were optimized. As the newly designed CrAssPFL2 combination showed human fecal specificity with higher detection efficiency than the other ones, this new primer/probe combination specifically targeting human-originated crAssphage and its optimized qPCR condition can be integrated to the current MST protocols for identifying human-derived fecal contamination.

## Data availability statement

The original contributions presented in the study are included in the article/Supplementary material, further inquiries can be directed to the corresponding author.

## Ethics statement

The study involving human participants was reviewed and approved by the IRB of Kyung Hee University (South Korea) to obtain human fecal samples. The approval number is KHGIRB-19-192. Written informed consent to participate in this study was provided by the participants or the participants’ legal guardian.

## Author contributions

S-YL and J-HL: conceptualization and methodology. S-YL and JHY: validation and investigation, data curation, and visualization. S-YL: resources. S-YL, JHY, and J-HL: writing original draft. JHY and J-HL: writing review and editing. J-HL: supervision and project administration and funding acquisition. All authors contributed to the article and approved the submitted version.

## Funding

This research was supported by Cooperative Research Program for Agriculture Science and Technology Development (Project no. PJ016298), Rural Development Administration, Republic of Korea.

## Conflict of interest

The authors declare that the research was conducted in the absence of any commercial or financial relationships that could be construed as a potential conflict of interest.

## Publisher’s note

All claims expressed in this article are solely those of the authors and do not necessarily represent those of their affiliated organizations, or those of the publisher, the editors and the reviewers. Any product that may be evaluated in this article, or claim that may be made by its manufacturer, is not guaranteed or endorsed by the publisher.

## Footnotes

^1^ https://www.ncbi.nlm.nih.gov/genbank/
