# Supplementary material for: Improvement of crAssphage detection/quantification method and its extensive application for food safety
Source: Front Microbiol. 2023 May 15;14:1185788. doi: 10.3389/fmicb.2023.1185788 (PMC10225732; doi:10.3389/fmicb.2023.1185788)
Supplement: Supplementary file 1 [file Data_Sheet_1.DOCX]

**Supplementary Information for**

**Improvement of CrAssphage Detection/Quantification Method and its Extensive Application for Food Safety**

**So-Young Lee^1,†^, Jihye Yang^2,4,5,†^, and Ju-Hoon Lee^2,3,4,5,*^**

*^1^ Department of Food Science and Biotechnology, Institute of Life Sciences and Resources,*

*Kyung Hee University, Yongin 17104, Korea*

*^2^Department of Agricultural Biotechnology, Seoul National University, Seoul 08826, South Korea*

*^3^ Department of Food and Animal Biotechnology, Seoul National University, Seoul 08826, South Korea*

*^4^ Center for Food and Bioconvergence, Seoul National University, Seoul 08826, South Korea*

*^5^ Research Institute of Agriculture and Life Science, Seoul National University, Seoul 08826, South Korea*

**^†^ These authors have contributed equally to this work and share first authorship**

*** Corresponding author:**

Dr. Ju-Hoon Lee / juhlee@snu.ac.kr

**Table S1.** The candidate qPCR compositions for optimization of crAssphage detection

| Composition | 1 | 2 | 3 | 4 | 5 | 6 | 7 | 8 | 9 | 10 |
| --- | --- | --- | --- | --- | --- | --- | --- | --- | --- | --- |
| Primer (μM) | 0.55 | 0.6 | 0.6 | 0.6 | 0.8 | 0.8 | 1 | 1 | 1.2 | 1.2 |
| Probe (μM) | 0.55 | 0.6 | 0.24 | 0.16 | 0.6 | 0.4 | 0.6 | 0.4 | 0.6 | 0.4 |
| Total volume (μl) | 25 | 25 | 25 | 25 | 25 | 25 | 25 | 25 | 25 | 25 |

**Table S2.** Cq values of qPCR for detection method optimization using each primer/probe combination

| Sample number^a^ | 1 | 2 | 3 | 4 |
| --- | --- | --- | --- | --- |
| CrAssBP | 29.81 | 22.06 | 24.37 | N.D.^b^ |
| CPQ056 | 29.83 | 28.77 | 24.24 | N.D. |
| RQ | 27.06 | 21.55 | 22.97 | N.D. |
| CrAssPFL1 | 36.88 | 21.03 | 23.19 | 28.71 |
| CrAssPFL2 | 27.64 | 22.49 | 22.47 | 30.01 |

^a^, Sample 1, 11-year-old male child’s fecal sample; Sample 2, 14-year-old male adolescents’ fecal sample; Sample 3, 12-year-old male adolescents’ fecal sample; Sample 4, 15-year-old male adolescents’ fecal sample.

^b^, N.D., Not detected

**Table S3.** Cq values of qPCR for sensitivity test using each primer/probe combination

| DNA concentration (ng/μl) | 10^0^ | 10^−1^ | 10^−2^ | 10^−3^ | 10^−4^ | 10^−5^ | 10^−6^ | 10^−7^ |
| --- | --- | --- | --- | --- | --- | --- | --- | --- |
| CrAssBP | 13.41 | 19.37 | 24.69 | 27.48 | 30.72 | 31.62 | 35.10 | N.D.^a^ |
| CPQ056 | 13.45 | 16.52 | 21.04 | 24.90 | 28.48 | 33.57 | 38.21 | N.D. |
| RQ | 10.61 | 13.41 | 17.12 | 21.36 | 24.30 | 28.16 | 31.28 | N.D. |
| CrAssPFL1 | 13.91 | 17.93 | 20.03 | 23.68 | 27.31 | 30.74 | 32.85 | 36.62 |
| CrAssPFL2 | 10.73 | 14.21 | 18.07 | 22.13 | 26.22 | 29.48 | 32.84 | 35.77 |

^a^, N.D., Not detected

**Figure S1.** Comparison of amplification between ten candidate qPCR compositions with each primer/probe set in the qPCR assay. Results of qPCR assay with primer/probe sets (A) CrAssBP, (B) CPQ056, (C) RQ, (D) CrAssPFL1, and (E) CrAssPFL2. The optimized composition for each crAssphage primer set in Table S1 was indicated with an arrow in the qPCR result graph.

**Figure S2.** qPCR results of CrAssBP, CPQ056, and RQ. qPCR with the primer/probe set (A) CrAssBP or (B) CPQ056 or (C) RQ was conducted to amplify DNA extracted from feces of each animal. Lanes M, 100 bp DNA ladder marker (MGmed); Lanes P, human fecal DNA; Lanes N, molecular water.

**FIGURE S1**

**A**

**
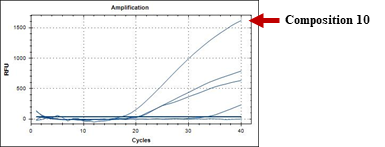
**

**B**

**
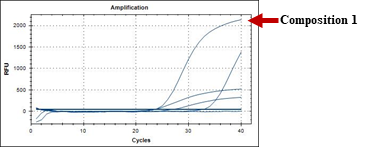
**

**C**

**
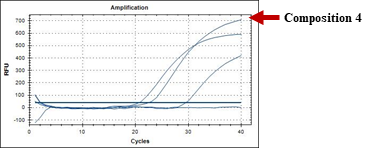
**

**D**

**
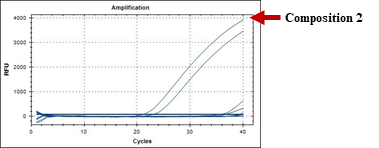
**

**E**

**
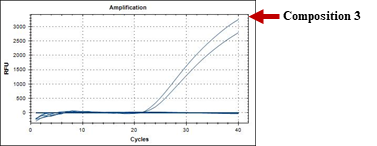
**

**FIGURE S2**

**
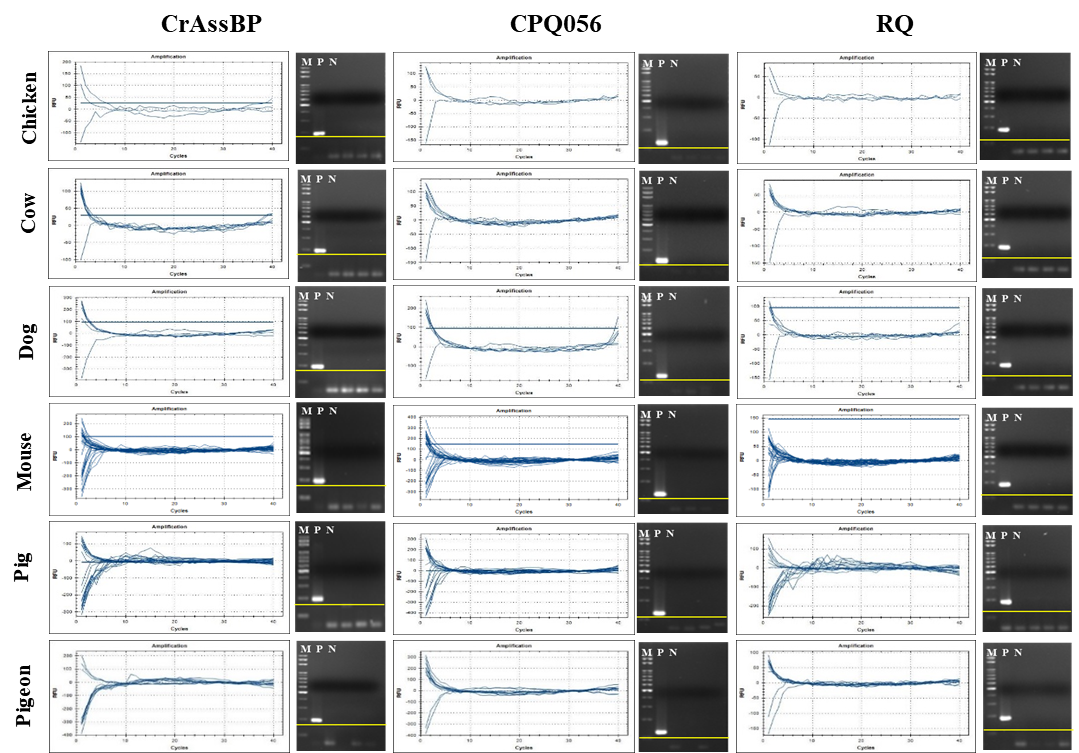
**
